# Outdoor PM_2.5_ concentration and rate of change in COVID-19 infection in provincial capital cities in China

**DOI:** 10.1038/s41598-021-02523-5

**Published:** 2021-12-01

**Authors:** Yang Han, Jacqueline C. K. Lam, Victor O. K. Li, Jon Crowcroft, Jinqi Fu, Jocelyn Downey, Illana Gozes, Qi Zhang, Shanshan Wang, Zafar Gilani

**Affiliations:** 1 Department of Electrical and Electronic Engineering, The University of Hong Kong, Pok Fu Lam, Hong Kong; 2 Department of Computer Science and Technology, The University of Cambridge, Cambridge, UK; 3 MRC Cancer Unit, Department of Oncology, The University of Cambridge, Cambridge, UK; 4 Department of Human Molecular Genetics and Biochemistry, Sackler Faculty of Medicine, Adams Super Center for Brain Studies and Sagol School of Neuroscience, Tel Aviv University, Tel Aviv, Israel

**Keywords:** Infectious diseases, Environmental impact, Risk factors

## Abstract

This study investigates thoroughly whether acute exposure to outdoor PM_2.5_ concentration, P, modifies the rate of change in the daily number of COVID-19 infections (R) across 18 high infection provincial capitals in China, including Wuhan. A best-fit multiple linear regression model was constructed to model the relationship between P and R, from 1 January to 20 March 2020, after accounting for meteorology, net move-in mobility (NM), time trend (T), co-morbidity (CM), and the time-lag effects. Regression analysis shows that P **(**β = 0.4309, *p* < 0.001) is the most significant determinant of R. In addition, T **(**β = −0.3870, *p* < 0.001), absolute humidity (AH) **(**β = 0.2476, *p* = 0.002), P × AH **(**β = −0.2237, *p* < 0.001), and NM **(**β = 0.1383, *p* = 0.003) are more significant determinants of R, as compared to GDP per capita **(**β = 0.1115, *p* = 0.015) and CM (Asthma) **(**β = 0.1273, *p* = 0.005). A matching technique was adopted to demonstrate a possible causal relationship between P and R across 18 provincial capital cities. A 10 µg/m^3^ increase in P gives a 1.5% increase in R (*p* < 0.001). Interaction analysis also reveals that P × AH and R are negatively correlated (β = −0.2237, *p* < 0.001). Given that P exacerbates R, we recommend the installation of air purifiers and improved air ventilation to reduce the effect of P on R. Given the increasing observation that COVID-19 is airborne, measures that reduce P, plus mandatory masking that reduces the risks of COVID-19 associated with viral-particulate transmission, are strongly recommended. Our study is distinguished by the focus on the rate of change instead of the individual cases of COVID-19 when modelling the statistical relationship between R and P in China; causal instead of correlation analysis via the matching analysis, while taking into account the key confounders, and the individual plus the interaction effects of P and AH on R.

## Introduction

COVID-19 was first reported in Wuhan, China in December 2019. Since then, more than 116-million infections have been reported, resulting in 2-million deaths globally.

Recent COVID-19 studies have investigated whether demography (D), co-morbidity (CM), meteorology, and lockdown have effects on viral infection^1–4^. Consistent with studies in SARS and MERS, depressed temperatures and rising humidity have been found to increase COVID-19 transmission^5,6^. Furthermore, influenza studies have suggested that exposure to PM_2.5_ (P) with and without interacting with meteorology may increase the risks of influenza infection^7^. In the US and Europe, chronic exposure to P and NO_2_ are linked to COVID-19 mortality^8,9^. Air pollution is considered to heighten the severity of COVID-19 infection, given that pollutants, such as P, may increase the risk of Vitamin-D deficiency and decrease immunity^10^. Increasingly, evidence suggests that air pollution is a significant contributor to COVID-19 infection^11–16^. Studies undertaken in China have concluded that P, NO_2_, and O_3_ associate with increased incidence of COVID-19 infections^17^, with significant interaction between air quality index (AQI) and rising temperature identified^18^. However, these studies have failed to fully account for the change in testing capacity and the inconsistency in COVID-19 case definition, as well as the confounding effects of D and CM. A few studies in Italy have explored the correlation relationship between the COVID-19 cases and the PM_2.5_ and PM_10_ levels without controlling potential confounders, such as mobility^19,20^. A more sophisticated and rigorous study recently conducted in Italy has utilized doubling-time derived from a fitted epidemic curve to measure COVID-19 transmission while reducing the noise of the observed data^21^. Without taking into account potential confounders, this study concludes that P alone does not facilitate COVID-19 transmission within the most affected regions^21^. However, one UK study has argued for a positive relationship between P and COVID-19 infection, after controlling for confounders, including population density, age, sex, diabetes, smoking-status, and cancer^22^. These indicate potential challenges in assessing acute P exposure effects on COVID-19 infection in China, given the existence of noise and irregularities underlying the epidemic trends, the lack of control of confounders to P exposure, and the lack of sophisticated models to control these data challenges. More rigorous statistical modelling and control methodologies are needed to reduce (1) the noise underlying the epidemic trends due to the lack of testing capacity and redefinition of confirmed cases, (2) the confounding biases that affect the causal link between P and COVID-19 infection, and (3) the collinearities across different meteorology, D, and CM variables.

In this study, we will examine the effect of P on the rate of change in the daily number of COVID-19 confirmed infections (R), across 18 high infection provincial capital cities in China, while addressing inadequacies in official case reporting due to the lack of testing capacity and inconsistencies in case definition, and taking into account confounders, including D, CM, meteorology, net move-in mobility (NM), time lag due to the incubation period, trends over time (T), and day-of-the-week (DOW) to reflect the recurrent weekly effect (see Table S5 in the Appendix for the definitions on the variables).

Outdoor P is chosen as the focus of our study given the assumption that R may be increased due to the potential deposition of viral droplets on P^23^. A recent rigorous study on COVID-19 aerodynamics has ascertained that viral aerosol droplets 0.25–1 µm in size can remain suspended in the air^24^. When such viral droplets are combined with suspending particles, P, they can travel greater distances, remain viable in the air for hours, and be inhaled deeply into the lungs, thus increasing the potential of airborne viral infection^25^.

Our study sheds new light on the effect of P in an outdoor environment, the interaction effect between P and absolute humidity (AH), and the effect of NM (lockdown), on R (the dependent variable). Our work reinforces the observation that COVID-19 droplets are airborne^24,26^, can suspend in the air and combine with the particulates, promoting infection via the airborne transmission pathway^27^.

## Results

### Descriptive statistics and data adjustment

**Figure 1:**
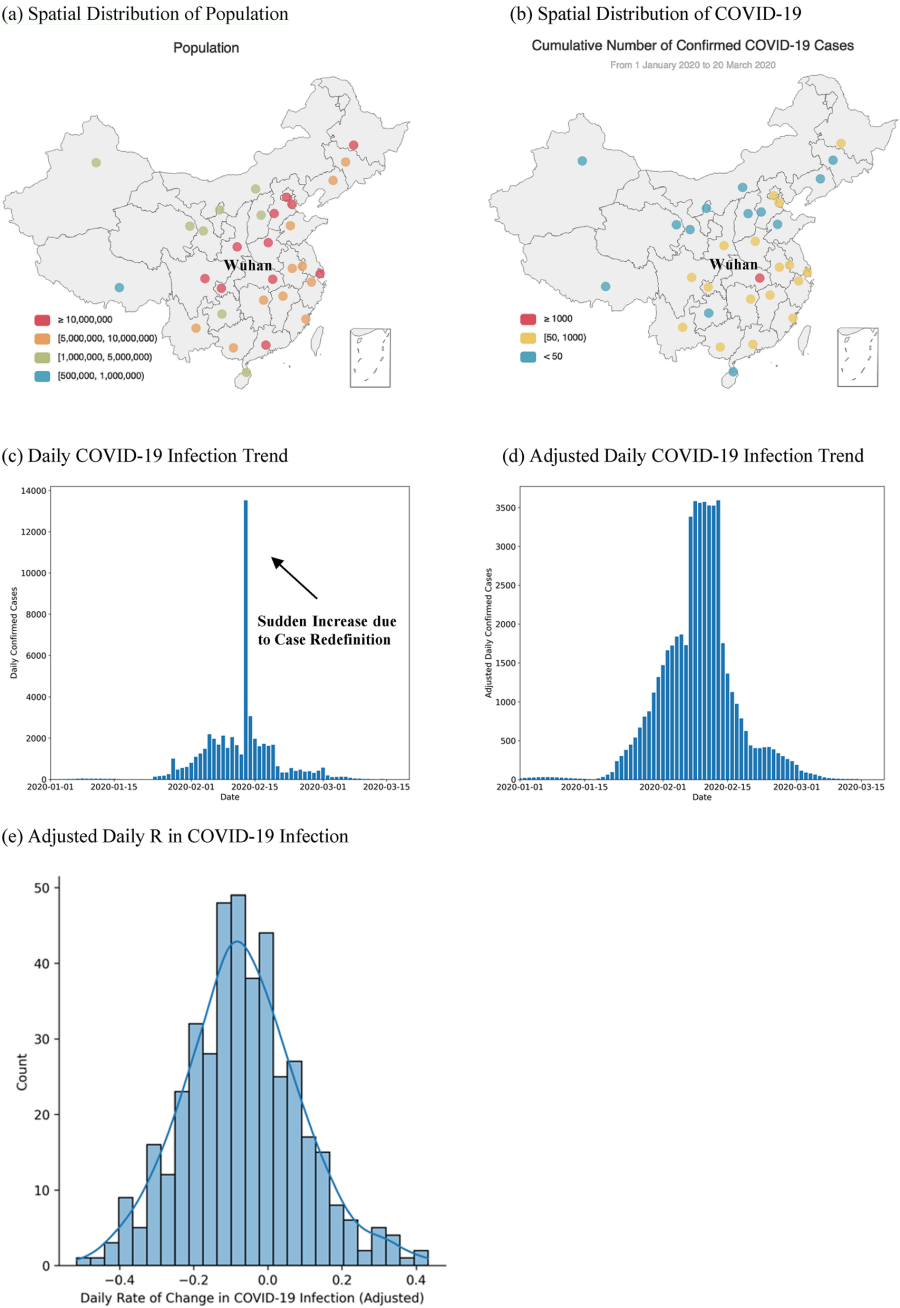
**(a)** Distribution of population across all provincial capital cities, **(b)** distribution of the cumulative number of confirmed COVID-19 cases across all provincial capital cities, **(c)** daily number of COVID-19 infection across all provincial capital cities, **(d)** adjusted daily number of COVID-19 infection across 18 high infection provincial capital cities, and **(e)** adjusted daily R in COVID-19 infection across 18 high infection provincial capital cities, China, from 1 January 2020 to 20 March 2020. The two maps in **(a,b)** were created by an open source Python library, pyecharts (version number: 1.9.0, URL: https://pyecharts.org/).

We collected data, including the number of confirmed COVID-19 cases, PM_2.5_ pollution, meteorology, mobility, demographics, and co-morbidities, in 31 cities in China, covering the period from 1 January to 20 March 2020 (see “Data collection and procedure” for more details). The spatial distributions of population and COVID-19 infection in these cities are shown in Fig. 1a,b. Collected COVID-19 infection data was pre-processed (see “Data pre-processing” for more details). 13 cities were removed due to small sample size (i.e., less than 50 confirmed cases in total). The remaining 18 cities (including Wuhan) were considered COVID-prone and were kept for further analysis. Due to potential delays in case reporting and redefinition of confirmed cases, COVID-19 infection data in 18 high infection provincial capital cities were adjusted by a moving average interpolation method and an outlier removal procedure, to reduce the short-term fluctuations in the reporting of COVID-19 confirmed cases and to recover the underlying epidemic trends (see “Data pre-processing”). Figure 1c,d highlight the trend of COVID-19 infection in China before and after data adjustment. Further, the adjusted daily confirmed cases were used to calculate R, a metric that measures relative percentage change in COVID-19 infection. R is derived from the difference between the number of COVID-19 infections of the current day and of the previous day, divided by the number of COVID-19 infections in the previous day (see “Data pre-processing”). By using R, even if the number of reported infections is inaccurate, relative changes in infection should be comparable (assuming consistent margins of error in case-reporting), with the adjusted data reflecting the underlying trends of COVID-19 infection. Figure 1e shows the adjusted distribution of R in 18 high infection provincial capital cities in China for the statistical analysis.

The sample data selection methodology is as follows: after data pre-processing, the number of data points became 412. For the 18 provincial capital cities, any city having more than 50 observations would then be selected. After adjusting the infection data, the rate of change in daily COVID-19 infections, R, was calculated, leading to a total of 1440 data points (80 days × 18 cities). 426 valid data points were obtained, given that many daily R values were unavailable due to (1) the number of infections at Day_*t*_ or Day_*t-1*_ were not reported (e.g., all cities except Wuhan have started reporting COVID-19 infections from late January 2020) and (2) zero infection was reported at Day_*t-1*_ (i.e., zero denominator). The adjusted R demonstrated a less significant Shapiro–Wilk normality test statistic (*W* = 0.994, *p* = 0.096), suggesting that the adjusted R followed a normal distribution. Finally, a few outliers were further removed according to the mean and the standard deviation of the R distribution, and 421 data points were obtained for statistical analysis. Four of the outliers were identified at the later stage of the epidemic, when the number of daily confirmed cases became minimal, despite some fluctuations. One outlier occurred around the time when the case definition in Wuhan was significantly changed and a sudden increase in the case reporting was observed. Therefore, removing these outliers can reduce the noise in the epidemic curve and improve the validity of the statistical results. The adjusted R ranged from -0.516 to + 0.432, after adjusting the infection data and removing the outliers.

### Statistical results

**Figure 2:**
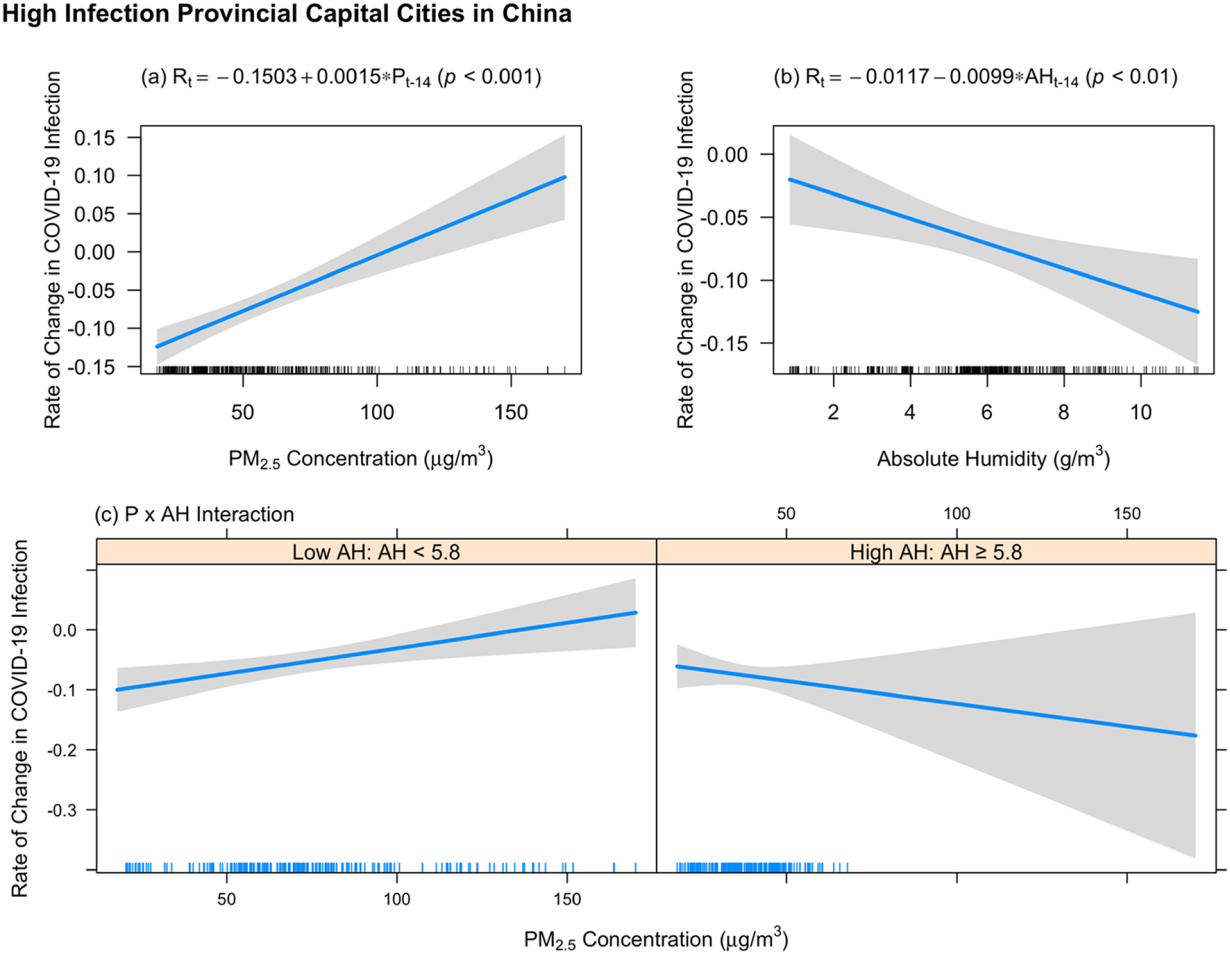
Significant P, AH, and P x AH determining R across 18 high infection provincial capital cities in China. **(a)** Univariate regression of significant P determining R, **(b)** univariate regression of significant AH determining R, and **(c)** interaction of significant P × AH determining R. The line (with confidence interval) in each plot represents the best-fit line that predicts R. The lines in the x-axis in each plot represent the observed data points.

After accounting for a one-day time-lag variable representing R of the previous day and the important confounding factors, including D, CM, meteorology, NM, and T, the best-fit stepwise regression model was constructed using data from all 18 high infection provincial capital cities in China. The results of the statistically significant independent variables (*p* < 0.05) that associate with dependent variable R, including their standardized coefficients (β), are shown in Table 1 (see “Statistical analysis” for more details). In order to illustrate the relationship between P, AH, and R across 18 high infection provincial capital cities in China, the univariate regression plots of P and AH are shown in Fig. 2a,b. To further illustrate the relationship between P × AH and R in China, AH is categorized by a cut-off point. The cut-off point is defined as the value when the partial derivative of the best-fit regression equation with respect to P is equal to zero, given that the variables other than P and AH remain unchanged (see Fig. 2c).

**Table 1:** Statistically significant independent variables that associate with dependent variable R across all 18 high infection provincial capital cities in China from 1 January to 20 March 2020.

Dependent variable: R_t_		Number of observations: *n* = 421	
Number of independent variables: 8		Adjusted R^2^: 41.15%	
Independent variable	Coefficient with 95% CI	Standardized coefficient (β)	*p*-value
Intercept	−6.846 × 10^–2^ (−1.970 × 10^–1^, 6.008 × 10^–2^)		0.2957
R_t−1_	2.510 × 10^–1^ (1.700 × 10^–1^, 3.319 × 10^–1^)	0.2725	2.52 × 10^–9^***
NM_t−L_	1.470 × 10^–2^ (5.133 × 10^–3^, 2.427 × 10^–2^)	0.1383	0.0027**
P_t−L_	2.208 × 10^–3^ (1.244 × 10^–3^, 3.173 × 10^–3^)	0.4309	8.77 × 10^–6^***
AH_t−L_	1.751 × 10^–2^ (6.243 × 10^–3^, 2.878 × 10^–2^)	0.2476	0.0024**
T_t_	−6.599 × 10^–3^ (−8.091 × 10^–3^, −5.108 × 10^–3^)	−0.3870	< 2*10^–16^***
GDP	5.545 × 10^–7^ (1.075 × 10^–7^, 1.001 × 10^–6^)	0.1115	0.0152*
Asthma	9.024 × 10^–4^ (2.677 × 10^–4^, 1.537 × 10^–3^)	0.1273	0.0054**
P_t−L_ × AH_t−L_	−3.779 × 10^–4^ (−5.903 × 10^–4^, −1.654 × 10^–4^)	−0.2237	0.0005***

1. P, AH, and NM are lagged and averaged by *L* = 14 days.

2. The standardized coefficient (also referred to as β coefficient) is calculated by multiplying the original regression coefficient by the ratio of the independent variable’s standard deviation to the dependent variable’s standard deviation.

3. *p-value < 0.05, **p-value < 0.01, ***p-value < 0.001.

As shown in Table 1, P, AH, NM, and T are the significant variables determining R across 18 high infection provincial capital cities in China. A higher P is associated with a higher R in China. When only observing the effect of P on R, a 10 µg/m^3^ increase in P is associated with a 1.5% increase in R (Coefficient = 0.0015, *p* < 0.001; see Fig. 2a). Moreover, AH is a significant variable accounting for R in China (Coefficient = 1.751 × 10^–2^, *p* = 0.002). As shown in Fig. 2b, when only observing the effect of AH on R, a higher AH decreases R. NM and T are also significant variables determining R in China. NM has a significant statistical correlation with R, and an increase in R is observed along with the increase in NM (Coefficient = 1.470 × 10^–2^, *p* = 0.003). T has a significant statistical correlation with R, and a decrease in R is observed along with the increase in T (Coefficient = −6.599*10^–3^, *p* < 0.001). Among D variables, GDP per capita is a significant variable that is positively associated with R (Coefficient = 5.545 × 10^–7^, *p* = 0.015). Among CM variables, asthma is a significant variable that is positively associated with R (Coefficient = 9.024 × 10^–4^, *p* = 0.005). The best-fit day-lag for P, AH, and NM is fourteen. Furthermore, based on the significant covariates identified in Table 1, the causal effect of P on R is established via matching, by addressing the confounding biases. The result is consistent with our main findings. On average, across 18 high infection provincial capital cities, according to the PM_2.5_ cut-off value set by China’s National Ambient Air Quality Standard^28^, in the days with a higher P (≥ 75 µg/m^3^) result in a 12.8% increase in R compared to the days with a lower P (< 75 µg/m^3^), after controlling for the important confounding factors including AH, NM, and T (see Table S6 in Appendix, p 8).

**Figure 3:**
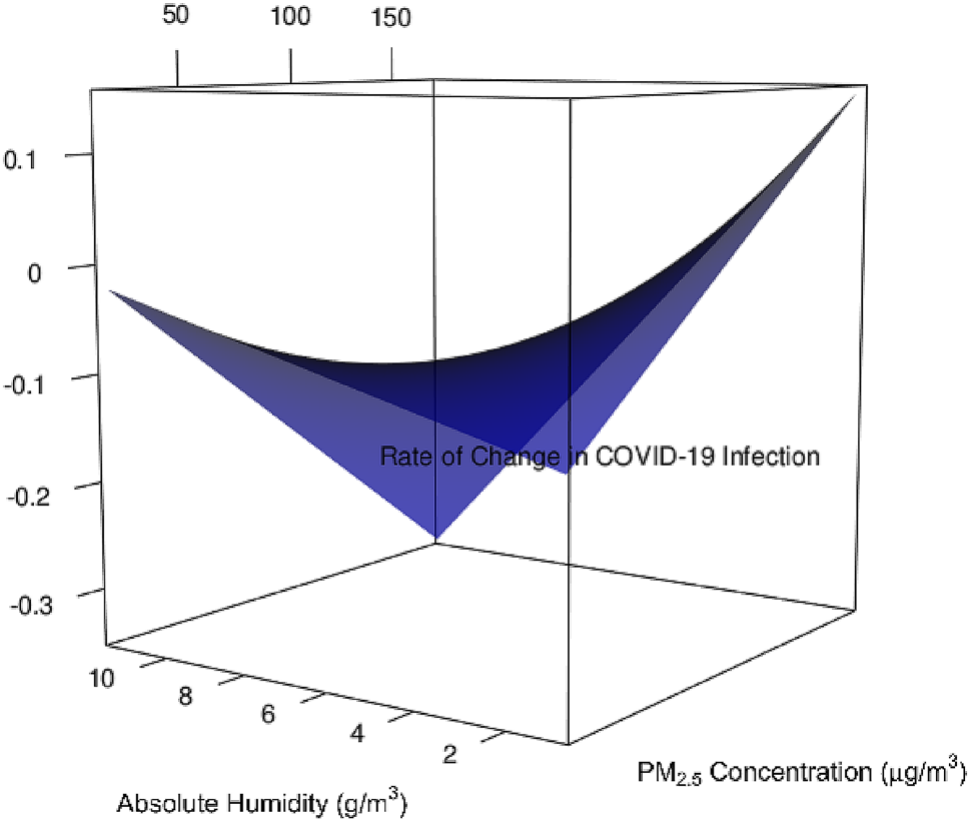
Relationship between P, AH, and R, based on the observed range of P and AH and the predicted R from the best-fit regression model, for 18 high infection provincial capital cities in China.

Moreover, the interaction between P and AH is significant across 18 high infection provincial capital cities in China (Coefficient = −3.779 × 10^–4^, *p* < 0.001; see Table 1). To further investigate the interaction between P and AH, AH was categorized into two levels according to the cut-off point of AH values when the partial derivative of the best-fit regression equation with respect to P is equal to zero. When AH is < 5.8 g/m^3^, a higher P and AH gave a higher R. When AH is ≥ 5.8 g/m^3^, a higher P and AH result in a lower R. As shown in the left part of Fig. 2c, when a higher P interacts with a lower AH, a higher R is still identified. In contrast, as shown in the right part of Fig. 2c, the effect of a higher P on R (in increasing trend) is counter-balanced by the effect of a higher AH on R (in decreasing trend). Nevertheless, based on the observed ranges of P and AH and the best-fit regression model that predicts R, a minimum R is attained when AH is 11.5 g/m^3^ and P is 170 µg/m^3^; whereas R (the rate of change in COVID-19 infection) is maximized when AH is 0.9 g/m^3^ and P is 170 µg/m^3^ (see Fig. 3).

Furthermore, when looking at the strength of the statistical relationship using standardized coefficient, for 18 high infection provincial capital cities in China, P, T, AH, P × AH, and NM are more important determinants of R (in descending order) when compared to D and CM, based on the values of β. Specifically, our regression analysis has shown that P **(**β = 0.4309, *p* < 0.001) is the most significant determinant of R, within the range of data collected for this study (see Table 1). T **(**β = −0.3870, *p* < 0.001), AH **(**β = 0.2476, *p* = 0.002), P × AH **(**β = −0.2237, *p* < 0.001), and NM **(**β = 0.1383, *p* = 0.003) are more significant determinants of R than D (GDP per capita) **(**β = 0.1115, *p* = 0.015) and CM (Asthma) **(**β = 0.1273, *p* = 0.005), but less significant than P (see Table 1).

Finally, when examining the multivariate normality assumption for the regression model listed in Table 1, results have shown that most data points in the normal quantile–quantile plot lie on the diagonal line (see Figure S2 in Appendix), though the Shapiro–Wilk normality test is more significant (*W* = 0.992, *p* = 0.016). Although the normality assumption may be violated, the regression model would still be able to generate valid results when the per variable sample size is sufficiently large (when the number is larger than ten)^29^.

## Discussion

Recent COVID-19 studies have investigated whether D, CM, meteorology, and lockdown affect viral infection and have ascertained that meteorological events can alter COVID-19 transmission^2^. Earlier studies have suggested that exposure to P can also increase influenza infection rates and identified PM_10_ and meteorological effects as risk-factors for SARS/MERS. In the US and Europe, long-term exposures to P and NO_2_ have been reported as the determinants of COVID-19 mortality, and evidence from China and Italy implicate air pollution as an attributor to COVID-19 infection. While previous research in China has concluded that P is associated with COVID-19 infection, it has yet to fully account for the changes in testing capacity, the inadequacy in confirmed case definition, and the confounding effects of D and CM. Recent studies have pointed towards the significant potential of COVID-19 transmission through the airborne pathway^30^.

To identify whether P affects R across 18 high infection provincial cities in China, including Wuhan, our regression model has accounted for all high potential confounders, including meteorological variables, NM, D at the provincial or city level, and CM at the provincial level, including eight major diseases that potentially decrease immunities and increase the risks of COVID-19 infection^31,32^. In addition, the time-lag effect on P, meteorology, and NM, have been addressed.

In particular, P with a lagged time of 14 days determines R, for all 18 high infection provincial capital cities in China, after accounting for the confounders/covariates. The higher the P value, the higher the R value. This implies that for one to reduce the COVID infection rate of change (R), the outdoor PM_2.5_ pollution concentration (P) across 18 Chinese provincial cities should be reduced. A 10 µg/m^3^ reduction in P will lead to 0.022 reduction in R after accounting for the covariates (see Table 1). Controlling and reducing outdoor P, and reducing the possibility for P to act as a carrier for COVID-19 viruses, require immediate public health attention. Public health measures such as installing air purifiers^33^, both indoors and outdoors, and improving air ventilation^34^, can help reduce P and reduce R. In particular, we recommend different methods of mechanical ventilation, including the installation of fans along with HEPA filters on the windows or within the air ducts to purify outdoor and ambient air. In this ventilation scheme, a slight negative pressure can be maintained to reduce the level of humidity and PM condensation, which in turn deters the viral load. If mechanical ventilation is less likely or not possible, then wind-driven natural ventilation is preferred for windows and other openings, alongside the use of pollution filters. Further, cross and stack ventilation will facilitate the smooth inflow of pollutant-free fresh clean air^35^. Moreover, putting P aside, given that AH and P x AH are important determinants of R, adjusting AH and P appropriately within a reasonable range (0 µg/m^3^ < P < 170 µg/m^3^ and 5.8 g/m^3^ < AH < 11.5 g/m^3^) can help reduce R substantially (see Fig. 3).

Further, NM and T are significant determinants of R in China. An increase in mobility within the provincial capital cities would increase R, whilst a decrease in mobility can reduce R. Finally, D and CM are less significant determinants of R when compared to P, AH, P × AH and NM. Having said so, GDP per capita is singled out as a significant D determinant of R whilst Asthma is singled out as a significant CM determinant of R. This implies that provincial cities having a higher GDP per capita (the more affluent cities) have a higher R (more infectious), whilst provincial cities having a higher burden of asthma (in DALY) are also more vulnerable to COVID-19 attacks, as asthma is often linked to airway inflammation and may increase COVID-19 susceptibility. Currently, only aggregate and annual D and CM data have been used for our regression analysis. Future study can make good use of D and CM data of higher temporal-spatial resolutions to provide us with better insights on how D and CM affect R across 18 high provincial capital cities in China.

Our model offers numerous advantages over those proposed in the previous literature covering air-pollution related COVID-19 epidemiological studies in four ways. First, instead of observing the absolute number of infections, which may be inaccurate due to possible human or systemic deficiency (related to testing methods and changes in case definition), our study examines R, the rate of change in COVID-19 infection (see “Data collection and procedure”). R can more sufficiently reflect the relative change in infection numbers, if the adjusted COVID-19 infection trends are consistent. Our focus on R instead of the actual infection number thereby provides much greater resolving power when compared to the previous air pollution and COVID-19 infection/mortality studies, which focus on the absolute number of infections^17,18^ instead^36^.

Second, our study addresses a wide spectrum of confounders that can affect observations concerning the effect of P on R, including key meteorological, NM, D, and CM variables. This stands in contrast to the existing works that explore the effects of air pollution on COVID-19 infection/mortality by controlling for only the meteorological variables^17,37^, or the meteorological variables and simple D variables, without considering the lockdown and CM variables^38^. Furthermore, while taking into account the confounding effects, our work also addresses the issues of non-linearity, collinearity, and time-lag (see “Data pre-processing”). This is particularly critical for precision modelling when (1) the statistical relationships between meteorology and R can be non-linear, (2) certain covariates among the meteorology, demographics, or co-morbidity variables can be collinear, and (3) the short-term effects of P, meteorology, and NM on R can be time-lagged due to the incubation period for COVID-19. By testing non-linearity and collinearity, and by accounting for the time-lag between some of our confounders and R, our model provides a more reliable and rigorous scientific explanation concerning how and when P will determine R across 18 high infection provincial capital cities in China, including Wuhan, in contrast to other prior air pollution-related COVID-19 infection/mortality models which have yet accounted for these confounding/covariate issues^8,38^.

Third, this is the first study that pursues the individual effects of P and AH on R, as well as the interaction effect of P and AH on R, covering 18 high infection provincial capital cities in China. Our study ascertains that a higher P increases R, and a higher AH decreases R. A 10 µg/m^3^ increase in P is associated with a 1.5% increase in R in China on average (*p* < 0.001; see Fig. 2a). Further, when P interacted with AH, their interaction effect on R is significantly negative (β = −0.2237, *p* < 0.001, see Table 1). After breaking down AH into two groups according to the optimal cut-off value, when AH is $<$ 5.8 g/m^3^, a higher P still leads to a higher R (see Fig. 2c). However, when AH is ≥ 5.8 g/m^3^, the effect of a higher P on R is counteracted by the effect of AH on R. When AH is 11.5 g/m^3^ and P is 170 µg/m^3^, a minimum R is achieved. When AH is 0.9 g/m^3^ and P is 170 µg/m^3^, a maximum R is achieved (see Fig. 3).

Finally, to the best of our understanding, this is the first international study that demonstrates a causal relationship between P and R across 18 high infection Chinese provincial capital cities, via matching. Each high P exposure day is matched with a low P exposure day sharing similar background covariates such as AH and NM to estimate the causal effect (Appendix p 8). This causal relationship between immediate P exposure and R (i.e. a higher P can increase R, see Table 1 and Table S6 in Appendix p 8), when combined with the recent reports that particulates less than 10 µm can facilitate the deposition of COVID-19 viral droplets and be suspended in the air^23^, further substantiates the recent observations concerning the risks of airborne infection^24,26^.

Although PM_2.5_ levels are low globally, they remain high in China. It has been estimated that the reduction of PM_2.5_ concentration due to lockdown during the specified period across the provincial capital cities of China is 9.7%, which remains small as compared to the 15.4% reduction of NO_2_ concentration^39^. With such reduction, PM_2.5_ level in most of these cities will still fail to meet the WHO standards. For example, the daily PM_2.5_ level in Shanghai is more than four times over the WHO threshold limit (10 µg/m^3^)^40^. The high PM_2.5_ concentration during the lockdown period confirms that the contribution of PM_2.5_ from the transportation sector is small, while the PM_2.5_ level generated from industrial production and residential coal combustion are much larger, and should be properly controlled if we want to reduce the PM_2.5_ level and hence COVID-infection in China^39^.

The COVID-19 transmission is primarily human-driven and the previous day infection along with human mobility are important factors for predicting R. Based on β, our results suggest that P is the most significant one predicting R during the first wave of COVID-19 in China, within the data range collected for this study (i.e., daily city-level PM_2.5_ concentration ranging from 2.6 μg/m^3^ to 208.4 μg/m^3^). Such findings are consistent with current studies that examine the effects of air pollution on R during the initial stage of outbreak. A cross-county study in US suggests that PM_2.5_ pollution is a more significant contributor to R during the early outbreak, when compared to population density^41^. A cross-country study suggests that PM_2.5_ is one of the most significant factors that associates with R during the early-stage outbreak across the world^42^. Non-pharmaceutical interventions that target to reduce human-to-human contact, such as school closure and stay-at-home order, are less significant as compared to R during the early-stage outbreak^42^. Nevertheless, when the number of COVID-19 infections reaches a certain threshold, the impact of PM_2.5_ on R is likely to be reduced to the minimal, when compared to factors such as the number of current infection cases.

All in all, increasing the risk of airborne COVID-19 viral infection is too high a cost to be ignored. Proper public health measures, such as mandating citizens to wear masks, are highly recommended to protect one from contracting COVID-19 via the viral-particulate transmission pathway, especially for countries of high population densities and mobilities, and high ambient particulate concentrations. Further, reducing the ambient PM_2.5_ particulate concentrations can substantially reduce the chance of COVID-19 infection. The installation of air purifiers and air ventilation improvement are recommended to reduce the effect of P on R. Meanwhile, after taking in account the number of days required for official reporting, given that the best fit linear regression model is yielded at the 14-day time-lag interval, P, AH, P × AH and NM values obtained 14 days prior to COVID-19 infection of the day can serve as the best determinants of R of the day. A 14-day time lag for best determining R suggests a 14-day incubation period is needed for any COVID-19 patient to become symptomatic in China, based on the COVID-19 data obtained during the first wave of COVID-19 infection in China. This shall serve as an important piece of public health information, regarding the number of days needed for quarantine for rigorous COVID-19 detection and control.

The current study presents certain limitations, which can be addressed in future studies: First, study that explore the causal relationship of the variables cannot be done properly when observational data with potential confounding biases are being used^43^. Spurious positive correlations are more likely found in non-stationary epidemiologic time series data^44^. The current study has incorporated the relevant confounders as much as possible and has adopted the matching method to further reduce the confounding effects. However, our preliminarily determined causal relationship may deserve further verification given that relevant epidemiological variables included in the regression model are yet to exhaustive. In the future, advanced causal inference techniques, such as instrumental variables estimation, can be used to further account for any unobserved confounding factors. Second, when analysing a wide variety of phenomena, it is possible to run into the look-elsewhere effect (also known as the multiple comparison problem)^45^. The current study adopts a stepwise regression approach in search of a set of significant variables for the best-fit model. The selection of significant variables involves multiple statistical tests and may be less robust due to the look-elsewhere effect^46^. In the future, bootstrap cross-validation techniques can be adopted to improve the robustness of model selection^47^. Finally, our study considers the incubation period as an interval ranging from 1 to 14 days, based on a uniform probability distribution. Given that the incubation period could have a more sophisticated distribution, more advanced statistical models using the Bayesian framework^48^ could be investigated to better account for the non-uniform distribution of the incubation period.

## Method

### Data collection and procedure

We collected data covering the daily P and the daily number of confirmed infections across 31 provincial capital cities in China, covering the period from 1 January to 20 March 2020 (see Fig. S1 in Appendix p 3). This was the period when COVID-19 infection was first officially announced in China, the lockdown measures were strictly exercised in Wuhan and other parts of China, and the number of confirmed cases peaked and dropped (see Fig. S1 in Appendix p 3). Other data at the provincial city-level were also collected on a daily basis (including meteorology and NM) or on a yearly basis (including D and CM) from internet sources and official statistical documents (see Table S1 in Appendix p 2). A full description of the dependent variable and the independent variables adopted for our statistical modelling is listed in Appendix (p 3–6). Descriptive statistics, including mean, standard deviation, minimum, and maximum values, are listed in Table S6 in Appendix. Given that the independent variables may not be normally distributed according to the Shapiro–Wilk normality test, the 25th percentile, the median (50th percentile), and the 75th percentile values are also reported in Table S6 in Appendix to better describe the distribution of variables. Table 2 highlights our research objectives and procedures.

**Table 2:** Research objectives and procedures.

Primary objective	1. Explore the statistical relationship, and determine the causal effects, if any, between daily outdoor P (PM_2.5_ concentration) and R (rate of change in daily COVID-19 infection) across the high infection provincial capitals in China, including Wuhan 2. To achieve this objective, we built two statistical models that can best address the following challenges in statistical analysis: (a) Redefinitions and potential delays in infection case reporting (b) Incubation period (c) Confounders and confounding biases, including meteorology, mobility/lockdown, demographic, co-morbidity, and time-trends (d) Collinearity (e) Linear relationship (f) Interaction between P and meteorology
Secondary objective	3. Highlight the conditions under which R can be reduced, and effective public health measures that can be employed to facilitate this 4. Add weight to the current observations that COVID-19 can be airborne and that particulates can be carriers of the viral droplets

### Data pre-processing

Earlier COVID-19 studies expressed reservations concerning the number of infection cases reported, given inadequate testing capacity, the change in confirmed case definition, and undiscovered and undocumented asymptomatic cases^3,49,50^. In order to address the delay in testing capacity and the change in case definition and their effects on reported cases, we used R, rate of change, as the dependent variable, in order to capture the relative change in COVID-19 infection during the study period. By using R, even if the number of reported infections might deviate, the relative change in infection could still be accounted for, provided that the reporting trends remain consistent.

Moreover, to remove the potential errors due to outliers and irregularities observed from the COVID-19 reported trends, a four-step data cleaning procedure was applied. First, 13 cities with a cumulative number of confirmed cases less than 50 were removed due to small sample size. This cut-off value was based the assumption that at least five types of independent variables should be taken into account in our model (including P, meteorology, NM, D, and CM) and that each independent variable requires at least ten samples for valid statistical analysis. As a result, only 18 high infection provincial capital cities had been selected for our statistical study. Second, for each city, to address the potential delay between the onset and the confirmation of COVID-19 infection, the adjusted daily confirmed COVID-19 infection cases were calculated by a rolling window of the observed daily confirmed cases reported in the following $W$ days (including the current day). The rolling window is a simple interpolation technique that smoothens the short-term fluctuations of the city-specific epidemic curve, while allowing for the backfill of delayed confirmed cases. More specifically, the adjusted number of confirmed cases on day $t$ was calculated as the average of the number of confirmed cases reported from day $t$ to $t+W-1$ (see Eq. 1).

1 $$\begin{array}{c}{\overline{N} }_{c,t}=\frac{{\sum }_{i=t}^{i=t+W-1}{N}_{c,i}}{W} \end{array}$$

where ${N}_{c,t}$ denotes the number of confirmed cases reported on day $t$ in city $c$. $W$ was set to 7 to address the reporting delay in COVID-19 case confirmation (which was estimated to be 7 days to 10 days^50^) and to account for the day-of-week fluctuations in case reporting. Further, any reported COVID-19 cases of zero value were removed, with the assumption that during the period of COVID-19 spread in China, the number of infection cases added per day would be greater than zero. Finally, for each selected city, daily R values were calculated throughout the study period (see Eq. 2).

2 $${R}_{c,t}= \frac{{\overline{N} }_{c,t}-{\overline{N} }_{c,t-1}}{{\overline{N} }_{c,t-1}}$$

where ${\overline{N} }_{c,t}$ denotes the number of adjusted confirmed cases reported on day $t$ in city $c$. For all R values across the selected cities, the mean and standard deviation of R were calculated. Assuming R follows a normal distribution, any R values out of the normal range (mean ± three times standard deviation) were considered as outliers and removed.

### Statistical analysis

We conducted statistical analysis in three steps. First, using stepwise multiple linear regression, a main effects model (i.e., without any interaction terms), including only the statistically significant variables in determining R, was constructed to model the relationship between daily outdoor P and daily R across 18 high infection provincial capital cities in China, while addressing the issues of collinearity and confounding brought by other independent variables. Second, to take into account the potential interaction effects between P and other significant meteorological and NM variables, the significant interaction terms were incorporated into the main effects model. A final regression models was developed for China (see Eq. 3).

3 $${R}_{c,t}={\alpha +{\beta }_{1}\text{ * }R}_{c,t-1}+ {{\beta }_{2}*P}_{c,t-L}+{{\beta }_{3}*AH}_{c,t-L}+{{\beta }_{4}*NM}_{c,t-L}+{\beta }_{5}*{T}_{t} +{\beta }_{6}*GDP+ {\beta }_{7}*Asthma+ {{\beta }_{8}*P \times AH}_{c,t-L}+\varepsilon$$

where $\alpha$ is the intercept, the subscript $c$ denotes a city, subscript $t$ denotes a day, subscript $L$ denotes the time lag for P, AH, and NM, and $\varepsilon$ serves as the error term. $L$ ranges from one to fourteen days. R denotes the rate of change in the daily number of confirmed COVID-19 infections. A one-day time-lag variable representing R of the previous day was also included in the model as an autoregressive term to account for the temporal auto-correlation among R time-series. P denotes the PM_2.5_ concentration. NM denotes the net move in mobility. AH denotes the absolute humidity. T is a variable representing the number of days since 1 January 2020, reflecting the time trend during the period of study. GDP represents gross domestic product per capita. Asthma represents the disability-adjusted life-year (DALY) numbers per 100,000 population. Two-sided *p*-values < 0.05 were considered significant for the statistical analysis. Third, the regression coefficient in the final regression model were standardized by multiplying the original regression coefficient by the ratio of the independent variable’s standard deviation to the dependent variable’s standard deviation, in order to compare the relative importance of each significant independent variable contributing to R.

Due to the lengthy asymptomatic incubation period before the onset of COVID-19 symptoms, the corresponding time-lag in P, meteorology, and NM was accounted for by our statistical analysis, using the multi-day average lag model, based on previous air-pollution related epidemiological studies^7^. We determined the best fit lag-time from day 1 to day 14, with the assumptions that the lag-time follows a uniform probability distribution and the mean incubation period could cover a maximum of 14 days^50^.

To estimate the causal effect of P on R, our model for China had to cover the potential confounders. Independent variables, including meteorology (AH, temperature (TEMP), air pressure (AP), and wind speed (WS)), and NM, were included in the statistical analysis for China as the confounders. Moreover, D (population density, age, sex, income, GDP per capita, and education) and CM (high blood pressure, diabetes, chronic obstructive pulmonary disease (COPD), stroke, obesity, asthma, Alzheimer’s disease (AD), and HIV/AIDS) were included in the statistical analysis to control for the provincial/city-level fixed effects in our model for China. T and day of week were included in the statistical analysis to control for the time-varying fixed effects and the recurrent fixed effects. The statistically significant variables were kept in the final fitted regression model. Furthermore, matching was adopted to further reduce the confounding biases in our model for China, by matching a high P day with a low P day, based on the similarities of corresponding confounders, thereby helping one more accurately estimate the causal relationship between P and R in China (see Appendix p 8).

To address the potential collinearity between the independent variables in our model for China, Spearman correlation analysis and variance inflation factor (VIF) analysis were performed. Before stepwise regression analysis, a Spearman correlation analysis was conducted to select a subset of variables that presented low collinearity in the meteorological, D, and CM data. The absolute Spearman Coefficient threshold was set to be 0.5 to detect the collinearity between any variables before the regression analysis, and to prevent the highly correlated variables from being included in the regression model^51,52^. First, AH and WS were selected as the meteorological variables for stepwise regression analysis. We tested the collinearity between TEMP, AP, WS, and AH, and removed TEMP and AP, due to their high collinearity with AH, which would be capable of accounting for the transmission of a flu virus^53^, and hence could also be used to account for R (|Spearman coefficient|> 0.5; see Table S2 in Appendix p 3). Second, population density, age (0–14 years old), age (> 65 years old), sex ratio (female/male), and GDP per capita were selected as the D variables for stepwise regression analysis. We tested the collinearity between D, population density, age (0–14 years old), age (> 65 years old), sex ratio (male/female), urban disposable income, GDP per capita, and education level (below high school). We found that all D variables, except for sex ratio and GDP per capita, correlated highly with population density and age. Population density and age might better account for R because (1) population density could account for the close-contact transmission of COVID-19^54,55^ and (2) old age could be linked to lower immunity^56,57^, making one more vulnerable to COVID-19 infection^58^. The correlation between population density and COVID-19 transmission was also ascertained in related studies conducted in Bangladesh and Italy^54,55^. Hence, urban disposable income and education level were removed, due to their high collinearity with population density and age (|Spearman coefficient|> 0.5; Table S3 in Appendix p 4). Third, high blood pressure, COPD, stroke, and asthma were selected as the CM variables for stepwise regression analysis. We tested the collinearity between CM variables, including high blood pressure, diabetes, COPD, stroke, obesity, asthma, AD, and HIV/AIDS. We found that all CM variables, except for stroke and asthma, correlated highly with high blood pressure and COPD, which were more common CMs identified from recent COVID infection cases, and might account for R^21^. Therefore, diabetes, obesity, AD, and HIV/AIDS were removed, due to their high collinearity with high blood pressure and COPD (|Spearman coefficient|> 0.5; Table S4 in Appendix p 4). Furthermore, after stepwise regression analysis, a variance inflation factor (VIF) analysis was performed to detect if any collinearity remained in the main effects model. An independent variable with VIF exceeding 10 was considered a high collinearity with other independent variables^51,59^. No collinearity was identified from the main effect model.

To account for the potential non-linear relationship between the meteorological variables and R, a second-order polynomial transformation was applied to the selected meteorological variables, including AH and WS, during stepwise regression analysis. In addition to the original meteorological variables, a quadratic term of each selected meteorological variable was included in stepwise regression model to address non-linearity. Based on the final stepwise regression model that achieved the best fit, we decided to use the first-order meteorological variables.

To examine the interaction effects between P and other significant meteorological and NM variables, three interaction terms consisting of the statistically significant variables were included in stepwise regression model for determining R. Three interaction terms, including P × AH, P × NM, and NM × AH, were added to the main effects model for China. P × AH, the statistically significant interaction term that associated with R, was included in the final stepwise regression model.

Finally, the multivariate normality assumption was examined by investigating the residuals of the main regression model shown in Eq. (3) via (1) a normal quantile–quantile plot and (2) a normality test statistic (Shapiro–Wilk normality test). In general, a linear regression model assumes that the model residuals (i.e., the errors between the observed and predicted values) are normally distributed. If (1) the data points in a normal quantile–quantile plot lie on a diagonal line and (2) a less significant *p*-value (*p* > 0.05) derived from the Shapiro–Wilk normality test is observed, the residuals can be assumed to follow a normal distribution.

### Preprint

This article was submitted to an online preprint archive^60^.

## Supplementary Information

Supplementary Information.

## Data Availability

The dataset used in this study will be made available upon request to the corresponding authors.

## Code availability

The data processing and statistical analysis code for this study will be made available upon request to the corresponding authors.
