# Supplementary material for: Outdoor PM2.5 concentration and rate of change in COVID-19 infection in provincial capital cities in China
Source: Sci Rep. 2021 Dec 1;11:23206. doi: 10.1038/s41598-021-02523-5 (PMC8636470; doi:10.1038/s41598-021-02523-5)
Supplement: Supplementary file 1 — Supplementary Information. [file 41598_2021_2523_MOESM1_ESM.docx]

**Outdoor PM_2.5_ Concentration and Rate of Change in COVID-19 Infection**

**in Provincial Capital Cities in China**

Yang Han^1, #^, Jacqueline CK Lam^1, #*^, Victor OK Li^1, #*^, Jon Crowcroft^2^, Jinqi Fu^3^, Jocelyn Downey^1^, Illana Gozes^4^, Qi Zhang^1^, Shanshan Wang^1^, and Zafar Gilani^1^

^1^Department of Electrical and Electronic Engineering, The University of Hong Kong, Pok Fu Lam, Hong Kong

^2^Department of Computer Science and Technology, The University of Cambridge, Cambridge, UK

^3^MRC Cancer Unit, Department of Oncology, The University of Cambridge, Cambridge, UK

^4^Department of Human Molecular Genetics and Biochemistry, Sackler Faculty of Medicine, Adams Super Center for Brain Studies and Sagol School of Neuroscience, Tel Aviv University, Israel

*Corresponding authors. Address: Department of Electrical and Electronic Engineering, The University of Hong Kong, Pok Fu Lam, Hong Kong. Email: [jcklam@eee.hku.hk](mailto:jcklam@eee.hku.hk); [vli@eee.hku.hk](mailto:vli@eee.hku.hk)

^#^The first three authors have equal contributions.

**Supplementary Appendix**

| **Table of Contents** | **Page** |
| --- | --- |
| Data Source | 2 |
| Study Scope | 2 |
| Variable Description and Summary Statistics | 3 |
| Regression Modelling | 7 |
| Estimating the Causal Effect of Immediate PM_2.5_ Exposure on Rate of Change in the Daily Number of Confirmed Infections | 8 |
| Statistical Analysis for Wuhan Only | 8 |
| References | 10 |

**Supplementary Material**

| **Abbreviation** | **Full form** |
| --- | --- |
| AD | Alzheimer’s disease |
| AH | Absolute humidity |
| AP | Air pressure |
| CM | Co-morbidity |
| COPD | Chronic obstructive pulmonary disease |
| D | Demography |
| DALY | Disability-adjusted life-year |
| DOW | Day of the week |
| NM | Net move-in mobility |
| P | PM_2.5_ |
| R | Rate of change in the daily number of confirmed COVID-19 infections |
| SEV | Summary exposure value |
| T | Time trend |
| TEMP | Temperature |
| WS | Wind speed |

**Data Source**

Data, including confirmed COVID-19 cases, PM_2.5_ (P), meteorology, and net move-in mobility (NM), were collected on a daily basis at the city level. Daily confirmed COVID-19 cases were collected from a popular online platform which aggregates the cases reported by the Chinese national/provincial health authorities for calculating the rate of change (R).^1^ P data were collected from the Chinese National Environmental Monitoring Center.^2^ The meteorological data, including temperature (TEMP), dew point, air pressure (AP), and wind speed (WS), were collected from the US National Oceanic and Atmospheric Administration (NOAA).^3^ The NM data, based on the move-in and move-out city movement index, was collected from Baidu, Inc.^4^ Other data including co-morbidity (CM) and demography (D) were collected on a yearly basis at the provincial city level from the following sources: CM data, including high blood pressure, diabetes, chronic obstructive pulmonary disease (COPD), stroke, obesity, asthma, Alzheimer’s disease (AD), and HIV/AIDS, were collected from relevant studies based on the Global Burden of Disease Study in 2017 and the China Hypertension Survey 2012-2015.^5,6^ D data, including population density (population/area), the percentage of population aged below 15 years old, the percentage of population with age above 65 years old, sex ratio (male/female), GDP per capita, urban disposable income, and the percentage of population who had not achieved a high school educational qualification, were all collected from the official Chinese statistical yearbooks.

**Table S1. Data source**

| **Data** | **Variable** | **Source** |
| --- | --- | --- |
| Confirmed COVID-19 cases | R | A popular online platform which aggregates the cases reported by the Chinese national/provincial health authorities^1^ |
| P | P | Chinese National Environmental Monitoring Center^2^ |
| Meteorology | TEMP, dew point, AP, and WS | US NOAA^3^ |
| NM | NM | Baidu, Inc.^4^ |
| D | Population density, the percentage of population with age below 15 years old, the percentage of population with age above 65 years old, sex ratio (male/female), urban disposable income, GDP per capita, and the percentage of population with education below high school | Chinese statistical yearbooks |
| CM | High blood pressure, diabetes, COPD, stroke, obesity, asthma, AD, and HIV/AIDS | Relevant studies based on the Global Burden of Disease Study in 2017 and the China Hypertension Survey 2012-2015^5,6^ |

**Study Scope**

We collected data covering the daily number of confirmed infections of 31 provincial capital cities in China, from 1 January to 20 March 2020. This was the period when COVID-19 infection was first officially announced in China, the lockdown measures were strictly exercised in Wuhan and other parts of China, and the number of confirmed cases peaked and dropped (see Figure S1). The 31 provincial capital cities of all provinces, autonomous regions, and municipalities in mainland China include Beijing, Changchun, Changsha, Chengdu, Chongqing, Fuzhou, Guangzhou, Guiyang, Haikou, Hangzhou, Harbin, Hefei, Hohhot, Jinan, Kunming, Lanzhou, Lhasa, Nanchang, Nanjing, Nanning, Shanghai, Shenyang, Shijiazhuang, Taiyuan, Tianjin, Urumqi, Wuhan, Xi’an, Xining, Yinchuan, and Zhengzhou (see Figure 1).^7^ After data pre-processing, the following 13 cities were removed due to their small sample size (i.e., less than 50 confirmed cases in total): Changchun, Guiyang, Haikou, Hohhot, Jinan, Lanzhou, Lhasa, Shenyang, Shijiazhuang, Taiyuan, Urumqi, Xining, and Yinchuan. The remaining 18 cities were considered the high infection provincial capital cities for further analysis.

(a) Daily Confirmed COVID-19 Cases in provincial capital cities (b) Daily Cumulative Confirmed COVID-19 Cases in provincial capital cities


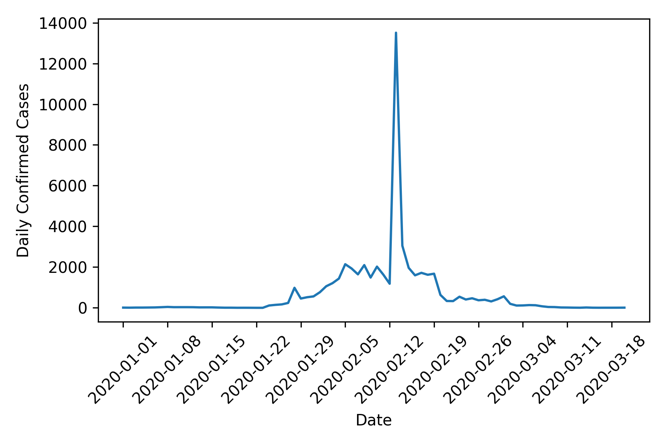

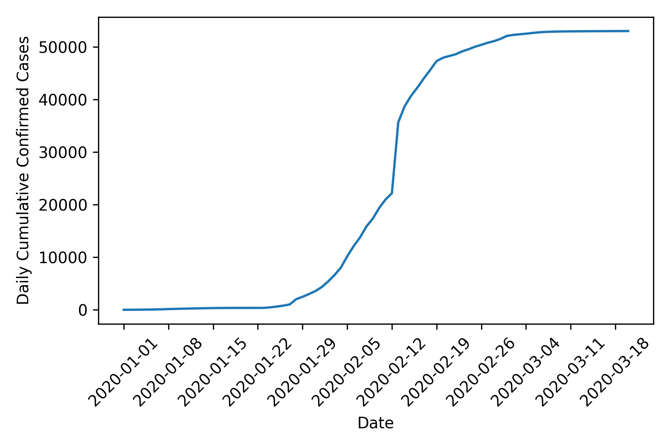


Lockdown in Wuhan

Sudden increase due to case redefinition

**Figure S1.** The first wave of COVID-19 in provincial capital cities in China, from 1 January 2020 to 20 March 2020

**Variable Description and Summary Statistics**

**COVID-19 Infection:** Daily COVID-19 infection was measured by R, based on the number of daily adjusted confirmed cases. R is defined in Eq (2) in the main text.

**PM_2.5_ Concentration:** Daily P was calculated as the mean of hourly P on that day from all monitoring stations of a city.

**Meteorological Condition:** Daily meteorology measurements were calculated as the mean of hourly meteorology measurements on that day from all monitoring stations of a city. Daily meteorology measurements that correspond to the same period of daily P were included as confounders in our regression modelling, including absolute humidity (AH), TEMP, AP, and WS. AH was converted from the corresponding dew point, TEMP, and AP. We focus on AH as it was previously reported to better explain the survival and transmission of influenza virus than relative humidity.^8^ Based on Spearman correlation analysis, there was a high collinearity across different meteorological variables, including AH, TEMP, and AP (|Spearman coefficient| > 0.5; see Table S2).

**Table S2. The Spearman correlation between PM _2.5_ and meteorology**

|  | P | AH | TEMP | AP | WS |
| --- | --- | --- | --- | --- | --- |
| P | - | - | - | - | - |
| AH | -0.31  (< 0.001) | - | - | - | - |
| TEMP | -0.30  (< 0.001) | **0.86**  (< 0.001) | - | - | - |
| AP | 0.05  (0.319) | **-0.52**  (< 0.001) | **-0.56**  (< 0.001) | - | - |
| WS | -0.26  (< 0.001) | -0.15  (0.002) | -0.12  (0.017) | 0.03  (0.560) | - |
| **Notes**  1. *p*-value in parenthesis. | | | | | |

**Net Move-in Mobility:** The effect of lockdown measures in Wuhan and the rest of the provincial capital cities in China can be measured by NM, calculated by subtracting the normalized move-out mobility index from the normalized move-in mobility index, respectively representing the number of people moving out of the city and moving into the city, downloadable from Baidu, Inc.^4^ Mobility indexes that correspond to the same period of daily P were examined (though these two indexes should be comparable across different cities, the details of how they are defined are not publicly available). Moreover, to capture the relative change in mobility during the Chinese New Year (25 January 2020) compared with the last Chinese New Year (5 February 2019), we deducted the mobility index recorded on a day in 2019 from the index recorded on the same day in 2020, based on the number of days before and after the Chinese New Year, which, traditionally, is the period of intensive movement in China.

**Demography and Co-morbidity:** To account for the variation across different cities in terms of their susceptibility to COVID-19, and to address the city-fixed effect, provincial or city-level D variables, including population density, age, sex, GDP per capita, income, and education, were included in our regression modelling (see Table S3 for more details). Based on Spearman correlation analysis, there were high collinearities among age (0-14 years old), urban disposable income, GDP per capita, and education level (below high school) (|Spearman coefficient| > 0.5; see Table S3). Moreover, provincial-level CM variables that might determine COVID-19 infection, including high blood pressure, diabetes, COPD, stroke, obesity, asthma, AD and HIV, eight major diseases that could potentially decrease the immunity system and increase the risk of COVID-19 infection,^9,10^ were included in our regression modelling (see Table S4 for more details). Based on Spearman correlation analysis, there were high collinearities among (1) high blood pressure, diabetes, and obesity and (2) COPD, AD, and HIV/AIDS (|Spearman coefficient| > 0.5; see Table S4).

**Table S3. The Spearman correlation between key demographics variables**

|  | Population density | Age (0-14 years old) | Age (> 65 years old) | Sex ratio | GDP per capita | Urban disposable income | Education level (below high school) |
| --- | --- | --- | --- | --- | --- | --- | --- |
| Population density | - | - | - | - | - | - | - |
| Age (0-14 years old) | 0.17  (< 0.001) | - | - | - | - | - | - |
| Age (> 65 years old) | -0.09  (0.066) | -0.27  (< 0.001) | - | - | - | - | - |
| Sex ratio | -0.04  (0.383) | -0.00  (0.985) | -0.48  (< 0.001) | - | - | - | - |
| GDP per capita | 0.22  (< 0.001) | -0.49  (< 0.001) | 0.05  (0.546) | 0.22  (< 0.001) | - | - | - |
| Urban disposable income | 0.25  (< 0.001) | -0.33  (< 0.001) | -0.03  (0.276) | 0.19  (0.001) | **0.88**  (< 0.001) | - | - |
| Education level (below high school) | 0.05  (0.281) | **0.74**  (< 0.001) | -0.18  (< 0.001) | -0.01  (0.891) | **-0.63**  (< 0.001) | **-0.62**  (< 0.001) | - |
| **Notes**  1. *p*-value in parenthesis. | | | | | | | |

**Table S4. The Spearman correlation between key co-morbidities of COVID-19 disease**

|  | High blood pressure | Diabetes | COPD | Stroke | Obesity | Asthma | AD | HIV/AIDS |
| --- | --- | --- | --- | --- | --- | --- | --- | --- |
| High blood pressure | - | - | - | - | - | - | - | - |
| Diabetes | **0.62**  (< 0.001) | - | - | - | - | - | - | - |
| COPD | -0.14 (0.003) | -0.19  (< 0.001) | - | - | - | - | - | - |
| Stroke | 0.40  (< 0.001) | 0.16  (0.017) | 0.45  (< 0.001) | - | - | - | - | - |
| Obesity | **0.76**  (< 0.001) | **0.60**  (< 0.001) | -0.38  (< 0.001) | 0.09  (0.060) | - | - | - | - |
| Asthma | -0.39  (< 0.001) | -0.32  (< 0.001) | 0.46  (< 0.001) | 0.33  (< 0.001) | -0.48  (< 0.001) | - | - | - |
| AD | -0.09 (0.062) | -0.22  (< 0.001) | **0.67**  (< 0.001) | 0.39  (< 0.001) | -0.27  (< 0.001) | 0.36  (< 0.001) | - | - |
| HIV/AIDS | -0.44  (< 0.001) | -0.40  (< 0.001) | **0.72**  (< 0.001) | 0.21  (< 0.001) | -0.42  (< 0.001) | 0.45  (< 0.001) | 0.30  (< 0.001) | - |
| **Notes**  1. *p*-value in parenthesis. | | | | | | | | |

**Time Trend and Weekly Effect:** Two time-related variables were introduced to account for the unobserved time trend (T), represented by the number of days since 1 January 2020, and the day-of-week (DOW) effect, represented by a categorical variable ranging from Monday to Sunday.

**Table S5. Definitions of the dependent and the independent variables of the regression model covering all 18 high infection provincial capital cities in China**

| **Variable** | **Description** | **Definition/Measurement** | **Unit** |
| --- | --- | --- | --- |
| **Dependent variable:** | | | |
| R_t_ | Daily city-level R | Defined in Eq (1). | NA |
| **Independent variables:** | | | |
| R_t-1_ | Daily city-level R lagged by one day |  | NA |
| P_t-L_ | Daily city-level P lagged by L days |  | ug/m^3^ |
| AH_t-L_ | Daily city-level AH lagged by L days |  | g/m^3^ |
| TEMP_t-L_ | Daily city-level TEMP lagged by L days |  | °C |
| AP_t-L_ | Daily city-level AP lagged by L days |  | Pa |
| WS_t-L_ | Daily city-level WS lagged by L days |  | m/s |
| NM_t-L_ | Daily city-level NM lagged by L days |  | NA |
| D (annual level*) | Population density (city level) | Ratio of population size to administrative area (km^2^). | 10^4^ persons/km^2^ |
|  | Age (0-14 years old) (provincial level) | Percentage of population with age below 15 years old. | % |
|  | Age (> 65 years old) (provincial level) | Percentage of population with age above 65 years old. | % |
|  | Sex ratio (city level) | Ratio of males to females. | NA |
|  | GDP per capita (city level) | Annual GDP per capita. | ¥ |
|  | Urban disposable income (city level) | Annual mean urban disposable income. | ¥ |
|  | Education level (below high school) (provincial level) | Percentage of population with education level below high school. | % |
| CM (annual provincial level) | High (systolic) blood pressure | Summary exposure value (SEV).^5^ | % |
|  | Diabetes | Disability-adjusted life-year (DALY) numbers per 100,000 population.^5^ | years/100,000 |
|  | COPD | DALY numbers per 100,000 population.^5^ | years/100,000 |
|  | Stroke | DALY numbers per 100,000 population.^5^ | years/100,000 |
|  | Obesity | The proportion of people having obesity.^6^ | % |
|  | Asthma | DALY numbers per 100,000 population.^5^ | years/100,000 |
|  | AD | DALY numbers per 100,000 population.^5^ | years/100,000 |
|  | HIV/AIDS | DALY numbers per 100,000 population.^5^ | years/100,000 |
| T | Continuous variable | The number of days since the starting date of the study period, 1 January 2020, indicating the time trend fixed effect for the unobserved change over time. | NA |
| DOW | Categorical variable | Indicating the day-of-the-week fixed recurrent effect. | NA |
| **Notes**  * For each type of D variables, city-level statistics were used if they are available for all 18 high infection provincial capital cities. Otherwise, provincial-level statistics were used. | | | |

**Table S6. Variable descriptive statistics**

**(a) All variables across all 18 high infection provincial capital cities**

| **Variable** | **Unit** | **Mean** | **Standard deviation** | **Min.** | **25th** | **50th** | **75th** | **Max.** |
| --- | --- | --- | --- | --- | --- | --- | --- | --- |
| R | NA | -0.068 | 0.158 | -0.516 | -0.172 | -0.077 | 0.028 | 0.432 |
| P | ug/m^3^ | 47.687 | 36.374 | 2.606 | 24.489 | 37.611 | 60.137 | 208.359 |
| AH | g/m^3^ | 5.915 | 2.940 | 0.589 | 4.081 | 5.666 | 7.206 | 17.711 |
| TEMP | °C | 6.541 | 7.367 | -23.140 | 3.917 | 7.704 | 10.814 | 22.158 |
| AP | Pa | 1024.231 | 5.567 | 1008.525 | 1020.575 | 1025.000 | 1027.740 | 1037.978 |
| WS | m/s | 2.455 | 1.301 | 0.544 | 1.459 | 2.108 | 3.075 | 7.455 |
| NM | NA | -1.844 | 2.596 | -14.197 | -2.962 | -1.120 | -0.341 | 5.165 |
| Population density | 10^4^ persons/km^2^ | 0.253 | 0.232 | 0.072 | 0.118 | 0.133 | 0.379 | 1.004 |
| Age (0-14 years old) | % | 0.156 | 0.037 | 0.098 | 0.137 | 0.164 | 0.181 | 0.219 |
| Age (> 65 years old) | % | 0.119 | 0.019 | 0.083 | 0.109 | 0.122 | 0.132 | 0.150 |
| Sex ratio | NA | 105.427 | 5.153 | 98.670 | 101.510 | 106.060 | 107.970 | 117.270 |
| GDP per capita | ¥ | 110455.35 | 31695.84 | 55513.00 | 85114.00 | 102037.00 | 136920.00 | 155491.00 |
| Urban disposable income | ¥ | 47781.00 | 10706.85 | 34889.00 | 39042.15 | 42988.00 | 59308.00 | 68034.00 |
| Education level (below high school) | % | 0.642 | 0.120 | 0.314 | 0.636 | 0.673 | 0.722 | 0.777 |
| High (systolic) blood pressure | % | 6.374 | 0.566 | 5.000 | 6.080 | 6.500 | 6.730 | 7.490 |
| Diabetes | years/100,000 | 703.361 | 100.571 | 547.450 | 673.960 | 685.460 | 753.510 | 955.500 |
| COPD | years/100,000 | 1445.478 | 629.319 | 640.300 | 1003.650 | 1318.960 | 1733.770 | 2960.450 |
| Stroke | years/100,000 | 2949.934 | 934.839 | 1350.910 | 1862.560 | 3084.450 | 3490.850 | 4775.060 |
| Obesity | % | 5.124 | 3.105 | 1.600 | 2.900 | 3.500 | 6.200 | 12.200 |
| Asthma | years/100,000 | 113.609 | 22.233 | 79.070 | 92.750 | 117.190 | 127.700 | 148.330 |
| AD | years/100,000 | 491.098 | 75.097 | 370.220 | 448.970 | 495.450 | 538.870 | 653.070 |
| HIV/AIDS | years/100,000 | 112.998 | 150.470 | 17.620 | 23.200 | 47.850 | 90.510 | 582.240 |

**(b) Key variables for each individual high infection provincial capital city**

| **Variable** | **Unit** | **Mean** | **Standard deviation** | **Min.** | **25th** | **50th** | **75th** | **Max.** |
| --- | --- | --- | --- | --- | --- | --- | --- | --- |
| **Beijing** | | | | | | | | |
| R | NA | -0.072 | 0.134 | -0.429 | -0.147 | -0.083 | 0.000 | 0.296 |
| P | ug/m^3^ | 69.412 | 61.838 | 3.892 | 23.401 | 49.207 | 112.190 | 208.359 |
| **Changsha** | | | | | | | | |
| R | NA | -0.059 | 0.157 | -0.222 | -0.185 | -0.080 | 0.008 | 0.300 |
| P | ug/m^3^ | 45.638 | 17.020 | 24.845 | 34.058 | 43.193 | 53.048 | 94.433 |
| **Chengdu** | | | | | | | | |
| R | NA | -0.082 | 0.147 | -0.375 | -0.150 | -0.091 | -0.021 | 0.270 |
| P | ug/m^3^ | 44.223 | 15.506 | 23.237 | 35.158 | 41.084 | 46.618 | 86.374 |
| **Chongqing** | | | | | | | | |
| R | NA | -0.093 | 0.122 | -0.438 | -0.135 | -0.064 | 0.000 | 0.080 |
| P | ug/m^3^ | 47.616 | 17.540 | 22.066 | 34.433 | 41.821 | 57.339 | 85.248 |
| **Fuzhou** | | | | | | | | |
| R | NA | -0.051 | 0.164 | -0.375 | -0.122 | -0.082 | 0.000 | 0.333 |
| P | ug/m^3^ | 19.214 | 11.410 | 2.606 | 8.944 | 18.449 | 27.090 | 41.549 |
| **Guangzhou** | | | | | | | | |
| R | NA | -0.079 | 0.149 | -0.273 | -0.199 | -0.109 | 0.000 | 0.295 |
| P | ug/m^3^ | 22.362 | 12.665 | 3.500 | 12.119 | 20.387 | 33.940 | 43.728 |
| **Hangzhou** | | | | | | | | |
| R | NA | -0.105 | 0.134 | -0.300 | -0.204 | -0.117 | -0.075 | 0.182 |
| P | ug/m^3^ | 30.048 | 17.380 | 5.695 | 19.936 | 27.189 | 35.248 | 70.519 |
| **Harbin** | | | | | | | | |
| R | NA | -0.006 | 0.215 | -0.467 | -0.091 | -0.013 | 0.101 | 0.432 |
| P | ug/m^3^ | 76.635 | 48.993 | 15.237 | 43.649 | 62.176 | 94.706 | 180.431 |
| **Hefei** | | | | | | | | |
| R | NA | -0.065 | 0.162 | -0.333 | -0.175 | -0.061 | 0.016 | 0.348 |
| P | ug/m^3^ | 43.855 | 27.320 | 11.974 | 19.130 | 30.000 | 66.639 | 94.657 |
| **Kunming** | | | | | | | | |
| R | NA | -0.030 | 0.162 | -0.323 | -0.120 | -0.074 | 0.038 | 0.286 |
| P | ug/m^3^ | 26.611 | 5.348 | 17.898 | 24.557 | 27.196 | 28.524 | 38.619 |
| **Nanchang** | | | | | | | | |
| R | NA | -0.079 | 0.148 | -0.385 | -0.137 | -0.088 | 0.312 | 0.231 |
| P | ug/m^3^ | 29.675 | 15.347 | 8.520 | 18.858 | 27.518 | 36.857 | 62.733 |
| **Nanjing** | | | | | | | | |
| R | NA | -0.039 | 0.167 | -0.429 | -0.091 | -0.014 | 0.055 | 0.179 |
| P | ug/m^3^ | 42.627 | 22.134 | 18.353 | 24.767 | 36.718 | 52.129 | 94.294 |
| **Nanning** | | | | | | | | |
| R | NA | -0.026 | 0.165 | -0.300 | -0.121 | -0.045 | 0.100 | 0.294 |
| P | ug/m^3^ | 34.029 | 30.103 | 6.922 | 11.972 | 23.747 | 46.259 | 123.380 |
| **Shanghai** | | | | | | | | |
| R | NA | -0.121 | 0.119 | -0.389 | -0.171 | -0.141 | -0.075 | 0.115 |
| P | ug/m^3^ | 40.110 | 20.157 | 13.203 | 23.804 | 34.672 | 53.720 | 78.046 |
| **Tianjin** | | | | | | | | |
| R | NA | -0.052 | 0.137 | -0.300 | -0.136 | -0.091 | 0.067 | 0.222 |
| P | ug/m^3^ | 70.469 | 55.911 | 7.500 | 27.044 | 47.209 | 103.995 | 203.916 |
| **Wuhan** | | | | | | | | |
| R | NA | -0.094 | 0.200 | -0.516 | -0.263 | -0.109 | 0.026 | 0.345 |
| P | ug/m^3^ | 40.244 | 19.001 | 9.101 | 27.345 | 39.388 | 49.396 | 90.475 |
| **Xi’an** | | | | | | | | |
| R | NA | -0.053 | 0.163 | -0.385 | -0.136 | 0.000 | 0.051 | 0.167 |
| P | ug/m^3^ | 96.881 | 34.260 | 44.547 | 68.589 | 95.714 | 129.102 | 157.684 |
| **Zhengzhou** | | | | | | | | |
| R | NA | -0.062 | 0.118 | -0.308 | -0.135 | -0.047 | 0.040 | 0.116 |
| P | ug/m^3^ | 68.619 | 33.001 | 9.218 | 49.168 | 66.528 | 92.199 | 140.838 |

**Regression Modelling**

The full equation with all relevant variables is shown in Eq (S1).

$$R_{c,t}\boldsymbol{=}{\alpha+\beta_{1}\text{ * }R}_{c,t-1}+ {\beta_{2}*P}_{c,t-L}+\vec{\beta_{3}}* \boldsymbol{M}_{c,t-L}+\vec{\beta_{4}}* \boldsymbol{M}_{c,t-L}^{2}+\beta_{5}*NM_{c,t-L}+{\vec{\beta_{6}}*\boldsymbol{D}}_{c}+{\vec{\beta_{7}}*\boldsymbol{CM}}_{c}+{{\beta_{8}*T}_{t}\text{ + }\vec{\beta_{9}}\text{ }*DOW}_{t} +\varepsilon Eq (S1)$$

where α is the intercept, the subscript *c* denotes a city, subscript *t* denotes a day, subscript *L* denotes the time lag for P, meteorology, and NM, and ε serves as the error term. *L* ranges from one to fourteen days. P denotes the PM_2.5_ concentration. M is the matrix representing the meteorology including AH, TEMP, WS, and AP. M^2^ is the matrix representing the quadratic term of AH, TEMP, WS, and AP. NM is the vector representing the net move-in mobility. D is the matrix representing the provincial/city-level demography. CM is the matrix representing the provincial-level co-morbidity statistics. T (a continuous variable) and DOW (a categorical variable) denote the unobserved time-varying effects across 18 high infection provincial capital cities, including the time trend fixed effect (such as variation of lockdown effects over time) and the day-of-the-week fixed effect (such as variation of lockdown effects from Tuesday to Sunday, compared to Monday).

The stepwise regression analysis was performed in three steps. First, a stepwise linear regression model (using both forward and backward selection) was used to fit the data and to identify the statistically significant determinants that associate with R. The best-fit regression model was selected based on Akaike information criterion (AIC).^11,12^ Second, the significant interactions between P and other significant determinants were included in the final stepwise regression model. Third, the regression coefficients of the final regression model were standardized to compare the relative importance of significant determinants contributing to R. Two-sided *p*-values < 0.05 were considered significant for the statistical analysis. All statistical analyses were performed using R (version 4.0.3) and R packages lm.beta 1.5-1, MASS 7.3-53, car 3.0-10, MatchIt 4.1.0, and visreg 2.7.0.

After the best-fit stepwise regression model was constructed, the multivariate normality assumption was examined by investigating the residuals of the regression model. Figure S2 shows the normal quantile-quantile (Q-Q) plot that visually examines the normality of the residuals. Most data points in the normal Q-Q plot lie closely on the diagonal line. However, the Shapiro-Wilk normality test for the residuals suggests a more statistically significant non-normal distribution (*W*=0.992, *p*=0.016).


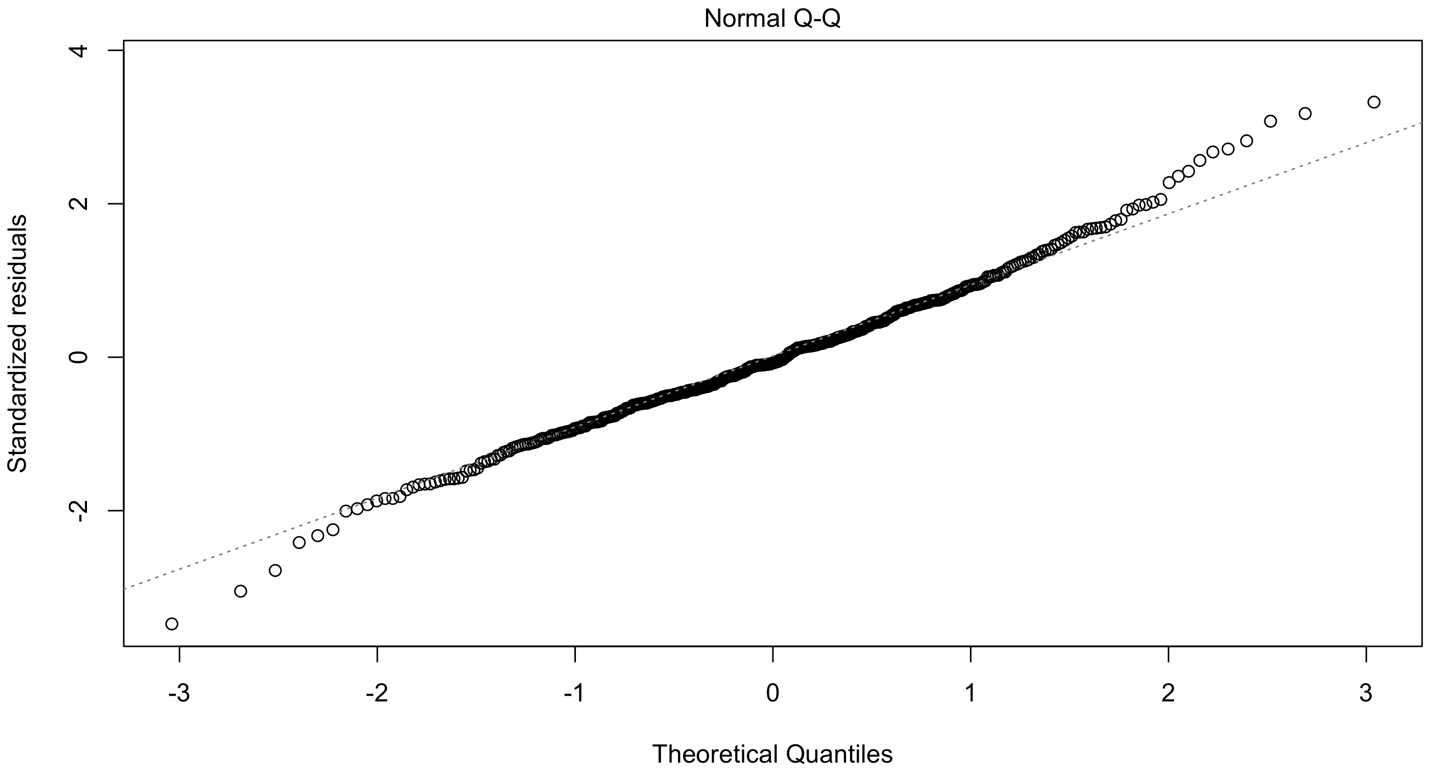
**Figure S2.** A normal Q-Q plot to examine the normality of the residuals

**Estimating the Causal Effect of Immediate PM_2.5_ Exposure on Rate of Change in the Daily Number of Confirmed Infections**

A matching approach^13^ was adopted to estimate the causal effect of immediate P exposure on R. First, each high P day (P ≥ 75 µg/m^3^) was matched with a low P day (P < 75 µg/m^3^) of similar background characteristics, sharing similar covariates, including NM, AH, and T (see Table 1). The cut-off value for P was determined according to the China’s Ambient Air Quality Standard. Next, the causal effect can be estimated based on the matched days. In particular, a logistic regression model was used to estimate the propensity score, where the dependent variable was the treatment status (i.e., high P=1; low P=0), and the independent variables were the significant confounding determinants including NM, AH, and T. Next, for each high P day, the control day was selected via the nearest neighbour matching with replacement. The nearest neighbour was determined by the estimated propensity score. Finally, utilizing the matched samples, a linear regression model was constructed based on Eq (1), but P was replaced by a binary variable indicating whether it was a high P day or not. We used matching to ensure an unbiased estimate of the causal effect, using a linear regression model. Two-sided *p*-values < 0.05 were considered significant for the statistical analysis. As shown in Table S7, when compared with the days of lower P (< 75 µg/m^3^), days of higher P (≥ 75 µg/m^3^; can be up to 170 µg/m^3^) could lead to a 12.8% increase in R on average.

**Table S7. The causal effect of P on R across 18 high infection provincial capital cities in China (from 1 January to 20 March 2020) based on matching**

| **Dependent Variable:** R_t_ | | **Number of Observations:** *n*=421 | |
| --- | --- | --- | --- |
| **Number of Independent Variables:** 8 | | **Adjusted R^2^:** 39.38% | |
| **Independent Variables** | **Coefficient with 95% CI** | | ***p*-value** |
| Intercept | 7.176 * 10^-2^ (-2.835 * 10^-2^, 1.719 * 10^-1^) | | 0.1596 |
| R_t-1_ | 2.733 * 10^-1^ (1.921* 10^-1^, 3.545 * 10^-1^) | | 1.14 * 10^-10^ *** |
| NM_t-L_ | 1.296 * 10^-2^ (3.004 * 10^-3^, 2.293 * 10^-2^) | | 0.0109 * |
| P_t-L_ (≥ 100 µg/m^3^) | 1.276 * 10^-1^ (3.821 * 10^-2^, 2.171 * 10^-1^) | | 0.0053 ** |
| AH_t-L_ | 6.655 * 10^-4^ (-6.573 * 10^-3^, 7.903* 10^-3^) | | 0.8567 |
| T_t_ | -6.650 * 10^-3^ (-8.142 * 10^-3^, -5.157 * 10^-3^) | | 2 * 10^-16^ *** |
| GDP | 4.807 * 10^-7^ (2.467 * 10^-8^, 9.368 * 10^-7^) | | 0.0389 * |
| Asthma | 7.091 * 10^-4^ (7.401 * 10^-5^, 1.344 * 10^-3^) | | 0.0287 * |
| P_t-L_ (≥ 100 µg/m^3^) × AH_t-L_ | -2.978 * 10^-2^ (-5.390 * 10^-2^, -5.666 * 10^-3^) | | 0.0156 * |
| **Notes**  1. P, AH, and NM were lagged and averaged by *L=*14 days.  2. P_t-L_ is a binary variable (0: P_t-L_ < 75 µg/m^3^; 1: P_t-L_ ≥ 75 µg/m^3^).  3. * *p*-value < 0.05, ** *p*-value < 0.01, *** *p*-value < 0.001 | | | |

**Statistical Analysis for Wuhan Only**

When only looking at Wuhan, the city with the greatest number of confirmed cases during our study period, the significant determinants in determining R were investigated. Following the same procedure in Section 4.3, the best-fit regression model for Wuhan was constructed (see Eq(S2)). Unlike the best-fit regression model for China, in the main effects model for Wuhan, AH was removed due to its high collinearity with other variables (variance inflation factor exceeding 10). Further, given that AH was removed and NM was not significant, no interaction term was added in the Wuhan model.

$$R_{\mathrm{wuhan},t}\boldsymbol{=} {{\alpha{+ \beta_{1}\text{ * }R}_{\mathrm{wuhan},t-1}+\beta}_{2}*P}_{\mathrm{wuhan},t-L}+\varepsilon Eq(S2)$$

The results of the statistically significant determinants that associate with R in Wuhan are shown in Table S8. The univariate regression plot of P and R is shown in Figure S2. P remains a statistically significant determinant of R (*p* < 0.01; see Table S8). When only observing the effect of P on R, a 10 µg/m^3^ increase in P is associated with a 10.8% increase in R in Wuhan (*p* < 0.001; see Figure S3). As compared to the final regression model for China, AH is not included in the final regression model for Wuhan due to high collinearity. Moreover, according to the stepwise regression analysis, NM (*p*=0.643) and T (*p*=0.327) are found to be less statistically significant associated with R in Wuhan, likely due to the fact that (1) the population movement from other cities to Wuhan, the epi-centre of the COVID-19 outbreak in China, during the outbreak period was minimal, and hence had generated very little impact on Wuhan, and (2) the population movement within Wuhan was constantly restricted due to the outbreak. Furthermore, based on the magnitude of the standardized coefficients, P is the most important contributor to R. Though the effect of P on R in Wuhan (β=0.4314, *p*=0.001, see Table S8) is similar to China (β=0.4309, *p* < 0.001; see Table 1), the model for Wuhan (adjusted R^2^ = 57.40%) fits the data better as compared to the model for China (adjusted R^2^ = 41.15%). This may be due to the paucity of data points at higher P in China (see Figure 2(a)), though P, AH, or P × AH are found to be more statistically significant determinants of R across 18 high infection provincial capital cities in China (see Table 1).

**Table S8.** Statistically significant determinants that associate with R in Wuhan from 1 January to 20 March 2020

| **Dependent Variable:** R_t_ | | **Number of Observations:** *n*=49 | | |
| --- | --- | --- | --- | --- |
| **Number of Independent Variables:** 2 | | **Adjusted R^2^:** 57.40% | | |
| **Independent Variable** | **Coefficient with 95% CI** | **Standardized Coefficient** | ***p*-value** |  |
| Intercept | -0.3574  (-0.5339,  -0.1808) |  | 0.0002 *** |  |
| R_t-1_ | 0.3205  (0.1257, 0.5153) | 0.4137 | 0.0018 ** |  |
| P_t-L_ | 0.0066  (0.0028, 0.0105) | 0.4314 | 0.0012 ** |  |
| **Notes**  1. P and AH are lagged and averaged by *L=*14 days.  2. D and NM are excluded in the initial stepwise regression model since they remained constant within the city.  3. The standardized coefficient (also referred to as beta coefficient) is calculated by multiplying the original regression coefficient by the ratio of the independent variable’s standard deviation to the dependent variable’s standard deviation.  4. * *p*-value < 0.05, ** *p*-value < 0.01, *** *p*-value < 0.001 | | | |  |

**
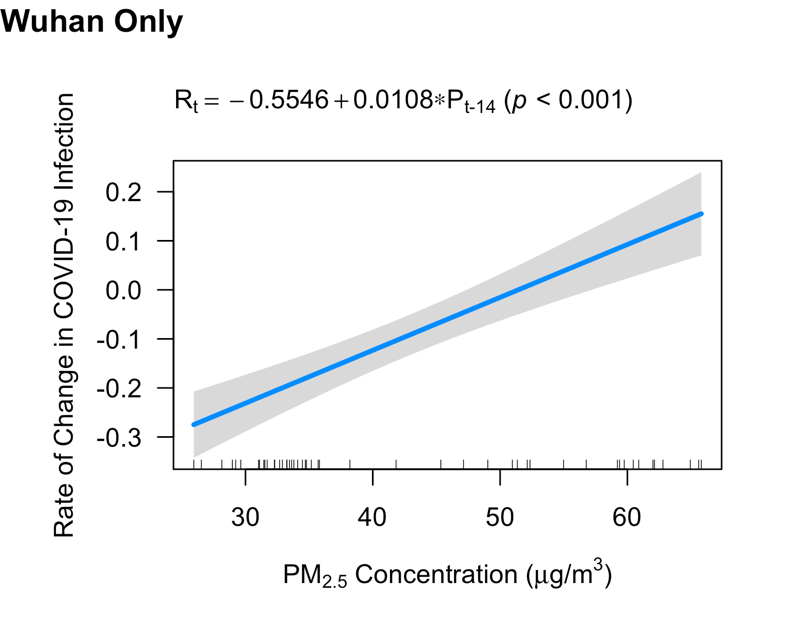
**

**Figure S3.** Univariate regression of significant P determining R in Wuhan

**References**

1 Xinxing Guanzhuang Bingdu Feiyan Yiqing Shishi Dongtai [Novel Pneumonia Real-Time Epidemic Information]. https://ncov.dxy.cn/ncovh5/view/pneumonia (accessed May 13, 2020).

2 Quanguo Kongqi Zhiliang Yubao Xinxi Fabu Xitong [China National Air Quality Reporting System]. http://106.37.208.228:8082/ (accessed May 13, 2020).

3 US NOAA. The Integrated Surface Database (ISD). https://www.ncdc.noaa.gov/isd (accessed May 13, 2020).

4 Baidu Qianxi [Baidu Mobility Map]. https://qianxi.baidu.com/ (accessed May 13, 2020).

5 Zhou M, Wang H, Zeng X, *et al.* Mortality, morbidity, and risk factors in China and its provinces, 1990–2017: a systematic analysis for the Global Burden of Disease Study 2017. *The Lancet* 2019; **394**: 1145–58.

6 Zhang L, Wang Z, Wang X, *et al.* Prevalence of overweight and obesity in China: Results from a cross-sectional study of 441 thousand adults, 2012–2015. *Obesity Research & Clinical Practice* 2020; **14**: 119–26.

7 Wang Y, Ying Q, Hu J, Zhang H. Spatial and temporal variations of six criteria air pollutants in 31 provincial capital cities in China during 2013–2014. *Environment International* 2014; **73**: 413–22.

8 Shaman J, Kohn M. Absolute humidity modulates influenza survival, transmission, and seasonality. *PNAS* 2009; **106**: 3243.

9 Yang J, Zheng Y, Gou X, *et al.* Prevalence of comorbidities and its effects in patients infected with SARS-CoV-2: a systematic review and meta-analysis. *International Journal of Infectious Diseases* 2020; **94**: 91–5.

10 Sattar Naveed, McInnes Iain B., McMurray John J.V. Obesity Is a Risk Factor for Severe COVID-19 Infection. *Circulation* 2020; **142**: 4–6.

11 Feng C, Li J, Sun W, Zhang Y, Wang Q. Impact of ambient fine particulate matter (PM2.5) exposure on the risk of influenza-like-illness: a time-series analysis in Beijing, China. *Environmental Health* 2016; **15**: 17.

12 Su W, Wu X, Geng X, Zhao X, Liu Q, Liu T. The short-term effects of air pollutants on influenza-like illness in Jinan, China. *BMC Public Health* 2019; **19**: 1319.

13 Baccini M, Mattei A, Mealli F, Bertazzi PA, Carugno M. Assessing the short term impact of air pollution on mortality: a matching approach. *Environmental Health* 2017; **16**. DOI:10.1186/s12940-017-0215-7.
